# Environmental beliefs and marine pro-environmental behaviors: mediating roles of responsibility and values among Chinese university students

**DOI:** 10.3389/fpsyg.2025.1710052

**Published:** 2025-12-12

**Authors:** Li Zhang, Lili Yang, Jiquan Zhou, Linzhao Wang

**Affiliations:** 1 College of Food Science and Technology, Guangdong Ocean University, Zhanjiang, Guangdong, China; 2 Mental Health Education and Counseling Center, Guangdong Ocean University, Zhanjiang, Guangdong, China

**Keywords:** general environmental beliefs, marine pro-environmental behaviors, marine environmental responsibility, marine environmental value, parallel mediation, Chinese university students

## Abstract

**Introduction:**

This study investigates the psychological mechanisms through which general environmental beliefs influence marine pro-environmental behaviors among Chinese university students, focusing on the parallel mediating roles of marine environmental responsibility and marine environmental value orientations.

**Methods:**

A crosssectional survey was conducted with 1,206 students from 23 universities across 11 provinces in China, using standardized instruments adapted from validated environmental psychology scales. Correlation analysis, hierarchical regression, and parallel mediation modeling with the PROCESS macro (Model 4) were employed to test the hypothesized independent mediation pathways.

**Results:**

General environmental beliefs significantly predicted pro-environmental behaviors both directly and indirectly. Responsibility and value orientations functioned as two independent and equally strong mediators, highlighting parallel psychological pathways. Neither gender nor age exerted significant effects, underscoring the predominance of psychological over demographic determinants.

**Discussion:**

Grounded in the Value–Belief–Norm theory and conceptually supported by the Theory of Planned Behavior and the Norm Activation Model, this study provides an integrated framework explaining how cognitive awareness translates into marine conservation behavior. The findings underscore the dual importance of normative obligation and value-based motivation in promoting youth engagement in ocean protection and suggest that marine education and policy design should incorporate strategies that strengthen both responsibility and value orientations.

## 1 Introduction

The ocean is the Earth’s largest ecosystem, covering over 70% of the planet’s surface and regulating climate, biodiversity, and human well-being through functions such as carbon sequestration, oxygen production, and nutrient cycling (Masterson-Algar et al., 2022). Yet, anthropogenic pressures are exerting unprecedented ecological pressures leading to ecological crises. According to the United Nations Environment Programme, approximately 8 million tons of plastic waste enter the ocean annually, endangering marine biodiversity and disrupting food chains (Oberoi and Garg, 2021). Overfishing has led to the depletion of about 33% of global fish stocks (Food and Agriculture Organization of the United Nations, 2020), while nearly 30% of anthropogenic carbon dioxide emissions are absorbed by the oceans, accelerating acidification and threatening coral reef ecosystems (Sabine et al., 2004). Such ecological degradation undermines biodiversity and jeopardizes economic stability and food security for coastal communities worldwide (Worm et al., 2006).

Recognizing the urgency of these issues, global governance frameworks such as the United Nations Convention on the Law of the Sea, the Convention on Biological Diversity, and the UN Decade of Ocean Science for Sustainable Development (2021–2030) emphasize evidence-based action and interdisciplinary collaboration (Ryabinin et al., 2019; IOC Executive Council and Intergovernmental Oceanographic Commission, 2018; Von Schuckmann et al., 2020). However, the effectiveness of these frameworks depends on individual and collective behavioral change, driven by psychological factors. Marine pro-environmental behaviors (MPEBs), such as reducing plastic use and supporting marine protected areas, remain underexplored compared to terrestrial behaviors, highlighting the need to investigate psychological drivers in diverse cultural contexts.

## 2 Theoretical framework and research status

### 2.1 Core constructs and multi-pathway model

Environmental psychology offers a robust framework for understanding pro-environmental behaviors (PEBs), integrating three core constructs: environmental beliefs, personal responsibility, and values, which form a multi-pathway model (Stern et al., 1999). General Environmental beliefs, commonly operationalized through the New Ecological Paradigm (NEP), reflect individuals’ awareness of ecological limits and human-nature interdependence, contrasting with the anthropocentric Human Exemptionalism Paradigm (HEP) (Dunlap and Van Liere, 1978). Strong NEP beliefs predict greater engagement in PEBs, such as supporting environmental policies (Lange and Dewitte, 2022).

Personal responsibility transforms beliefs into moral obligations, driving PEBs like recycling and energy conservation (Clayton, 2020). Values—egoistic, altruistic, and biospheric—shape environmental priorities, with biospheric and altruistic values strongly linked to sustained PEBs (Dietz et al., 2005). The Value-Belief-Norm (VBN) theory posits that values activate beliefs and responsibility, forming a causal chain to PEBs (Stern, 2000). The Theory of Planned Behavior (TPB) complements this by emphasizing attitudes, social norms, and perceived behavioral control (Ajzen, 1991). Meta-analyses report moderate to strong correlations between beliefs and PEBs (Steg, 2023). Emotional connectedness to nature, such as through the Connectedness to Nature Scale, further enhances motivation by boosting well-being (Mayer and Frantz, 2004). This multi-pathway model integrates VBN, TPB, and emotional factors. It provides a comprehensive lens for analyzing marine pro-environmental behaviors (MPEBs).

### 2.2 Psychological mechanisms of marine pro-environmental behaviors

Marine environments, characterized by limited visibility and perceived psychological distance, pose unique challenges to marine pro-environmental behaviors, such as reducing plastic use, supporting marine protected areas, and participating in beach cleanups (O'Halloran, 2025). Unlike terrestrial issues, marine problems (e.g., plastic pollution, overfishing) are often perceived as distant, reducing urgency and behavioral engagement (Kelly et al., 2022). Positive beliefs about marine ecosystem health significantly enhance marine pro-environmental intentions (McKinley et al., 2023). Emotional attachment to marine environments amplifies personal responsibility, fostering actions like refusing single-use plastics (Pahl et al., 2017).

The VBN framework applies to marine contexts, with studies showing that beliefs about plastic pollution’s harm trigger moral responsibility, increasing marine pro-environmental behavioral intentions (Wang et al., 2025b). Cross-national research indicates that perceived human-ocean interconnectedness strengthens responsibility (Chilvers et al., 2014). However, existing research on marine pro-environmental behaviors has been largely conducted within North American and European cultural frameworks, while studies in other cultural settings remain limited, where local traditions and institutions may shape distinct psychological pathways (Salazar-Sepúlveda et al., 2023). For instance, Jefferson et al. (2021) found that community-based marine conservation initiatives enhance marine pro-environmental behaviors through collective responsibility, suggesting a need for culturally tailored models.

### 2.3 Integrated theoretical framework: linking VBN, TPB, and NAM

From the VBN theory, values (egoistic, altruistic, and biospheric) form the motivational foundation that activates environmental beliefs and moral responsibility, establishing a value–cognition–normative chain that drives behavioral commitment. The NAM extends this pathway by emphasizing how moral obligations and internalized personal norms activate feelings of responsibility once individuals perceive the adverse consequences of environmental degradation and their ability to mitigate them (Yue et al., 2020). The TPB, in turn, contributes the social-cognitive dimension, highlighting that beliefs about outcomes, subjective norms, and perceived behavioral control shape behavioral intentions (De Leeuw et al., 2015).

The present study integrates three complementary theoretical perspectives—the Value–Belief–Norm (VBN) theory (Stern, 2000), the Theory of Planned Behavior (TPB) (Ajzen, 1991), and the Norm Activation Model (NAM) (Schwartz, 1977)—to construct a cohesive framework explaining marine pro-environmental behaviors (MPEBs) among Chinese university students. Each theory highlights a distinct but interrelated psychological component that underlies pro-environmental decision-making. Integrating these perspectives, the proposed model conceptualizes beliefs as the cognitive and attitudinal foundation derived from TPB, responsibility as the moral–normative component grounded in NAM, and values as the motivational basis from VBN. Together, these constructs represent cognitive, normative, and motivational pathways converging on MPEBs. This integration allows a nuanced understanding of how cognitive appraisals (beliefs) translate into moral commitment (responsibility) and are sustained by deep-seated value orientations.

### 2.4 Chinese university students and marine governance

China, with over 14,000 km of coastline, faces urgent marine sustainability challenges, including plastic pollution and overfishing (Chen and Martens, 2022). Under “marine power” and “ecological civilization” strategies, university students, as educated future leaders, are pivotal in advancing marine governance (Ministry of Education of the People’s Republic of China, 2020). However, Chinese students exhibit an intention-behavior gap, expressing concern for marine issues but engaging in limited marine pro-environmental behaviors due to low awareness and psychological distance (Wang et al., 2025a).

Recent global advancements in ocean literacy (OL) initiatives have underscored their importance in fostering environmental awareness and sustainable behaviors, particularly among youth and university students. For instance, Mallick et al. (2023) found that Hong Kong university students exhibit moderate levels of marine environmental knowledge and pro-environmental attitudes, influenced by academic majors and engagement in marine-related activities. Similarly, Kao et al. (2025) reported that Taiwanese college students possess fundamental marine ecological knowledge but demonstrate limited policy literacy and practical engagement skills, revealing a gap between environmental awareness and behavioral implementation. Chen and Tsai (2016) further highlighted that direct experiences with the marine environment can significantly enhance Taiwanese students’ pro-environmental behaviors, and therefore advocated for experiential and multimodal educational approaches. In the mainland Chinese context, Umuhire and Fang (2016) further demonstrated that educational interventions effectively improved university students’ ocean environmental awareness and responsibility, highlighting the need for culturally tailored marine education approaches. Beyond East Asia, Brennan et al. (2019) demonstrated that systems-based learning and simulation tools can improve marine knowledge, attitudes, and behaviors. Choi et al. (2024) further linked higher ocean literacy in South Korea to stronger climate change mitigation behaviors, while McHugh et al. (2024) highlighted the success of social marketing programs, such as the Explorers Education Programme, in broadening youth engagement. Despite these achievements, persistent challenges remain, including limited policy literacy, insufficient long-term evaluations, and regional disparities in ocean literacy initiatives (Kao et al., 2025; McHugh et al., 2024; Tsai et al., 2023). Addressing these limitations calls for integrating interdisciplinary curricula, experiential learning, and policy-oriented education to strengthen ocean literacy’s role in global ocean governance.

**Figure 1:**
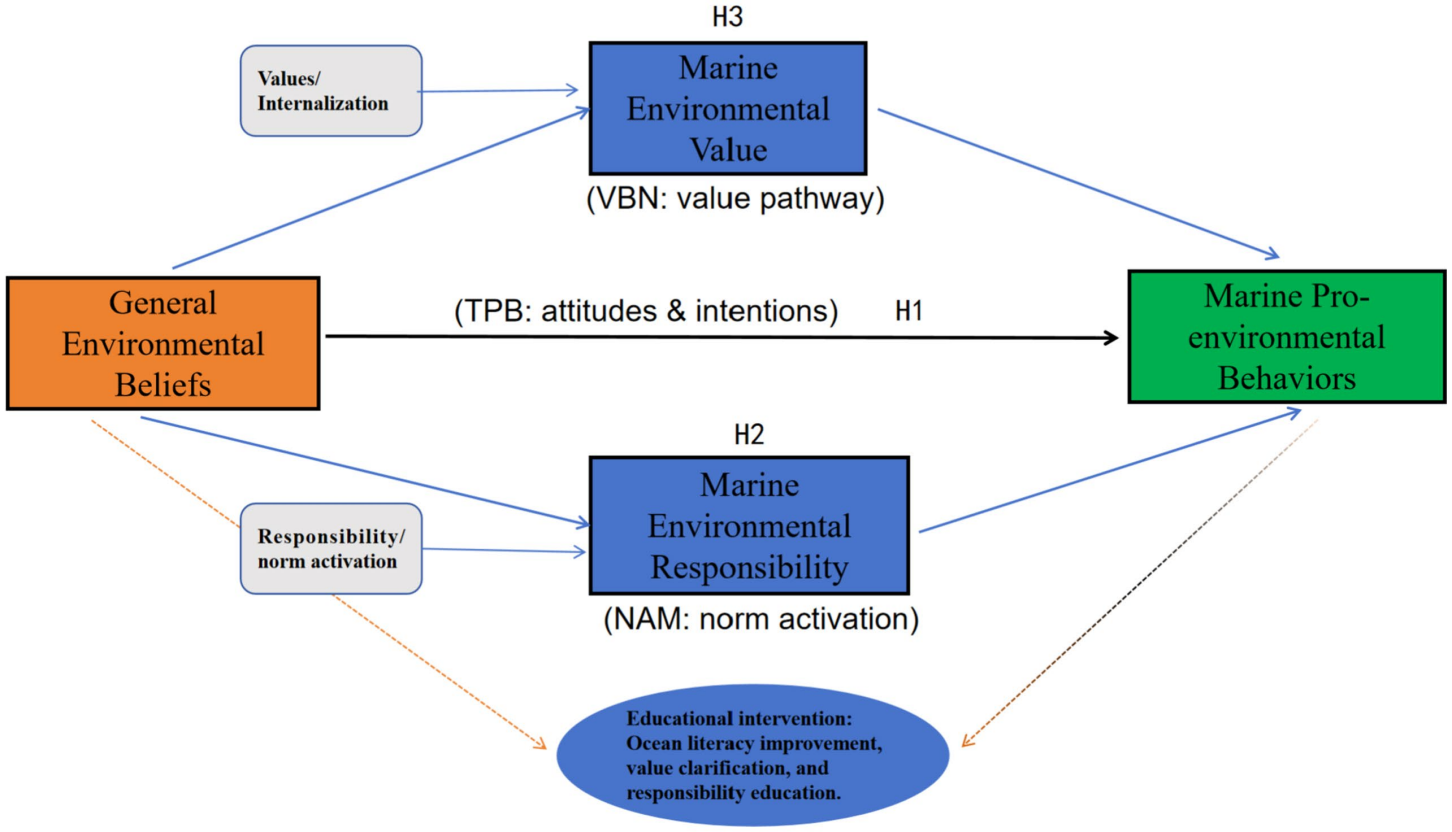
Conceptual model based on an integration of VBN, TPB, and NAM frameworks.

Building on the global progress of ocean literacy (OL) initiatives, this study advances a theoretical model explaining how psychological mechanisms shape marine pro-environmental behaviors among Chinese university students. Specifically, this study synthesizes the Value–Belief–Norm (VBN) theory (Stern, 2000), the Theory of Planned Behavior (TPB) (Ajzen, 1991), and the Norm Activation Model (NAM) (Schwartz, 1977) into an integrated multi-pathway framework (Figure 1). Within this framework, general environmental beliefs (NEP) serve as a central cognitive mechanism linking individuals’ values, moral responsibility, and behavioral engagement. The model conceptualizes three interconnected pathways through which pro-marine behaviors emerge: (1) a value-driven pathway, in which biospheric and altruistic values stimulate pro-marine beliefs and moral responsibility (VBN);(2) a cognitive–attitudinal pathway, where beliefs and perceived social expectations jointly shape behavioral intentions (TPB); (3) a normative–moral pathway, in which personal responsibility and moral obligation are activated and translated into concrete marine pro-environmental behaviors (NAM).

This integrative perspective extends previous single-theory approaches by revealing how cognition, morality, and motivation operate synergistically to transform environmental awareness into conservation-oriented action. In the Chinese collectivist context, the influence of social norms and collective responsibility tends to amplify the NAM component, while the state-led initiatives of moral education and “ecological civilization” reinforce the value-oriented mechanism of the VBN pathway (Hofstede, 2001; Tam and Chan, 2017). Accordingly, the hypothesized model (Figure 1) depicts these three interlinked routes as culturally embedded psychological mechanisms that explain how Chinese university students internalize marine environmental norms and commit to sustainable marine practices.

Moreover, marine place attachment—defined as individuals’ emotional and cognitive bond with marine environments—enhances pro-environmental attitudes by fostering empathy and stewardship motivations (Mayer and Frantz, 2004; Li et al., 2021). Empirical studies further support these associations; for instance, Wang et al. (2025b) demonstrated that marine education in China significantly strengthened environmental values and moral responsibility, thereby promoting pro-environmental behavioral intentions. Globally, although ocean literacy initiatives have expanded in scope and impact, persistent knowledge–action gaps remain—particularly in policy literacy, governance awareness, and long-term behavioral engagement. Addressing these challenges calls for educational innovation grounded in psychological theory. Educational interventions—such as marine-related courses, experiential learning (e.g., beach clean-ups), and virtual reality simulations—can effectively mitigate the intention–behavior gap by enhancing environmental beliefs, sense of responsibility, and value internalization (Chen et al., 2025).

Accordingly, the present study adopts a cross-sectional design to examine how general environmental beliefs, responsibility, and values jointly predict university students’ pro-environmental behaviors. By situating this investigation within China’s sociocultural and educational context, the study contributes to both theoretical integration and practical innovation. Future research will employ structural equation modeling and intervention-based validation to further substantiate these pathways and inform sustainable behavioral change.

### 2.5 Research objectives and hypotheses

Building on environmental psychology theory and prior empirical research, the present study proposes a model linking general environmental beliefs, responsibility, and value orientations to marine pro-environmental behaviors among Chinese university students. Specifically, we hypothesize:

*H1*: General environm ental beliefs positively predict pro-environmental behaviors.

*H2*: Marine environmental responsibility mediates the relationship between general environmental beliefs and pro-environmental behaviors.

*H3*: Marine value orientations mediate the relationship between general environmental beliefs and pro-environmental behaviors.

*H4*: The mediating effects of marine environmental responsibility and value orientations are comparable in strength, indicating independent yet equally important mechanisms.

## 3 Materials and methods

### 3.1 Participants and procedure

This study employed a cross-sectional survey design to examine the mediating roles of general environmental beliefs, sense of marine responsibility, and values in predicting pro-environmental behaviors. Participants were recruited through collaborative partnerships with 23 universities across 11 provinces in China between June and July 2024. Data were collected online using Wenjuanxing^1^, China’s largest and most widely used online survey platform, functionally comparable to Google Forms and widely applied in behavioral and educational research (e.g., Dai et al., 2023; Su et al., 2024). Wenjuanxing allows researchers to design and distribute questionnaires efficiently, provides broad population coverage, and ensures data quality through built-in IP restriction, real-time monitoring, and automatic response validation.

Given the wide geographic distribution of the target population, an online survey was chosen over face-to-face or telephone methods to enhance accessibility, representativeness, and cost-effectiveness. The online format also facilitated participant anonymity and minimized social desirability bias, which is particularly important in studies relying on self-reported environmental attitudes and behaviors. The questionnaire was uploaded with logic jumps, mandatory fields, and response-time checks, and a pilot test with 50 students refined wording and timing. Coordinators distributed unique survey links or QR codes via official WeChat groups, bulletin boards, and email lists. Response rates were monitored in real time, and the survey closed automatically upon reaching target quotas or at the end of July 2024. Raw data were exported with built-in validity filters (e.g., excluding overly rapid or uniform responses) to ensure data integrity.

The final sample included 1,206 valid responses out of 1,301 distributed questionnaires (effective response rate = 92.7%). Among the 23 participating universities, 21 were located in coastal provinces—including four marine-specialized institutions (Guangdong Ocean University, Dalian Ocean University, Jiangsu Ocean University, and Zhejiang Ocean University)—and two were in inland provinces (Henan and Hubei). These universities represent regions with varying levels of marine economic engagement and educational orientation, thereby enhancing the ecological validity of the findings. Participants were 46.1% male (*n* = 556) and 53.9% female (*n* = 650), aged 18–24 years (*M* = 20.39, SD = 1.42). All participants provided informed consent prior to participation. Table 1 presents the demographic characteristics of the sample.

**Table 1:** Characteristics of participants (*N* = 1,206 respondents).

Category	Variable	*N*/(%)
Age groups	18 and under	121(10%)
	19–22 years	1,007(83.5%)
	23 years and above	78(6.5%)
Gender	Male	556(46.1%)
	Female	650(53.9%)
Type of college	Coastal Colleges	700(58%)
	Inland Colleges	506(42%)
Degree of relevance to marine studies	Highly Relevant	242(20.1%)
	Moderately Relevant	201(16.7%)
	Not Relevant	763(63.2%)
Professional background	Humanities	231(19.2%)
	Science	286(23.7%)
	Engineering	428(35.5%)
	Medicine	88(7.3%)
	Other	173(14.3%)
Proximity to the Ocean	Coastal areas (near the sea)	806(66.8%)
	Mid-sea areas	204(17%)
	Offshore areas (far from the sea)	196(16.2%)

### 3.2 Measurement instruments

All instruments used in this study were adapted from well-validated scales in the fields of environmental psychology and education. To ensure their applicability among Chinese university students, a pilot study (*n* = 80) was conducted to evaluate cultural adaptability, clarity of wording, and psychometric properties. Based on participant feedback, necessary modifications were made to optimize the content and structure of the scales. Cronbach’s *α* coefficients were computed to evaluate the internal consistency of each scale. The present study examined how general environmental beliefs influence marine pro-environmental behaviors through the mediating roles of values and responsibility. All instruments were pretested and refined to ensure reliability and validity (see Table 2). The specific measures were as follows:

**Table 2:** Item wordings for the general environmental beliefs scale, marine environmental values, marine environmental responsibility, and marine pro-environmental behaviors scales.

Scale	No.	Items
General Environmental Beliefs (NEP) Scale	1	We are approaching the limit of the number of people the Earth can support.
	2	Humans have the right to modify the natural environment to suit their needs.
	3	When humans interfere with nature, it often produces disastrous consequences.
	4	Human ingenuity will ensure that we do not make the Earth uninhabitable.
	5	Humans are seriously abusing the environment.
	6	The Earth has plenty of natural resources if we just learn how to develop them.
	7	Plants and animals have as much right as humans to exist.
	8	The balance of nature is strong enough to cope with the impacts of modern industrial nations.
	9	Despite our special abilities, humans are still subject to the laws of nature.
	10	The so-called “ecological crisis” facing humankind has been greatly exaggerated.
	11	The Earth is like a spaceship with very limited room and resources.
	12	Humans are destined to rule over the rest of nature.
	13	The balance of nature is very delicate and easily upset.
	14	Humans will eventually learn enough about how nature works to control it.
	15	If things continue on their present course, we will soon experience a major ecological catastrophe.
Marine Environmental Responsibility Scale	1	My behavior affects the health of the marine environment.
	2	I am capable of protecting the marine environment.
	3	I can learn how to improve the marine environment.
	4	I will make efforts to improve the marine environment around me.
Marine Environmental Value Scale	1	Having rights is important to me.
	2	Wealth is important to me.
	3	Social status matters to me.
	4	Being influential is important to me.
	5	Maintaining social justice is important to me.
	6	Helping vulnerable groups is essential.
	7	Fairness is important to me.
	8	A world free from war and conflict is extremely important.
	9	Protecting the marine environment is important.
	10	Preventing marine pollution is important.
	11	Respecting and protecting the ocean is important.
	12	Living in harmony with nature is important.
Marine Pro-Environmental Behaviors Scale	1	I always pick up litter on the beach.
	2	I actively take actions in daily life to protect the marine environment.
	3	I reduce water use to lessen pressure on the marine ecosystem.
	4	I am willing to donate to marine protection organizations.
	5	I am willing to participate in activities organized by marine protection organizations.
	6	I would sign a petition for addressing marine environmental issues.
	7	I participate in school or community marine protection activities.
	8	The health of the ocean is vital to human survival.
	9	My actions can significantly affect the health of the ocean and coastal areas.
	10	I have a personal responsibility to work for ocean and coastal health.
	11	Citizens should take responsibility for ocean sustainability.
	12	I am willing to reduce household energy consumption.
	13	I avoid products harmful to marine life or derived from endangered species.
	14	I choose plastic-free alternatives whenever possible.
	15	I try to shorten my shower time.

#### 3.2.1 General environmental beliefs (NEP) scale

General environmental beliefs were measured using the revised New Ecological Paradigm (NEP) scale developed by Dunlap and Van Liere (1978). This decision was made to examine the influence of general environmental worldview on marine-specific variables. The instrument consists of 15 items across five dimensions: limits to growth, anti-anthropocentrism, fragility of nature’s balance, rejection of exemptionalism, and the possibility of an ecological crisis. Responses were rated on a 5-point Likert scale (1 = strongly disagree, 5 = strongly agree). The total Cronbach’s *α* in this study was 0.892, indicating good reliability and applicability in environmental psychology research (Chen and Martens, 2022).

#### 3.2.2 Marine environmental values scale

Environmental values were assessed using the scale developed by Xu (2008), based on Stern and Dietz’s (1994) value orientation framework. The scale contains 12 items across three dimensions: egoistic, altruistic, and biospheric values. Responses were rated on a 5-point Likert scale (1 = strongly disagree, 5 = strongly agree). The total Cronbach’s *α* was 0.955, with reliability coefficients of 0.896, 0.927, and 0.979 for the three dimensions, respectively, suggesting excellent reliability and construct validity.

#### 3.2.3 Marine environmental responsibility scale

Marine environmental responsibility was measured with an adapted version of Yue et al.’s (2020) Environmental Responsibility Scale, revised for the marine context. The instrument includes 4 items, each rated on a 5-point Likert scale (1 = strongly disagree, 5 = strongly agree). The Cronbach’s α was 0.887, demonstrating good reliability and validity.

#### 3.2.4 Marine pro-environmental behaviors scale

Marine pro-environmental behaviors was measured using a revised version of existing environmental behavior scales (Gong, 2008; Paredes Coral et al., 2022), adapted to capture the specific attributes of the marine environment. The scale consists of 15 items rated on a 5-point Likert scale (1 = strongly disagree, 5 = strongly agree). It comprises two dimensions (conservation-oriented, activism-oriented), with higher scores indicating stronger tendencies toward marine pro-environmental behaviors environmental behavior. Reliability testing demonstrated excellent internal consistency (Cronbach’s α = 0.96), confirming the scale’s strong reliability and validity.

### 3.3 Data analysis

Quantitative analyses were conducted using IBM SPSS Statistics 26.0. Descriptive statistics were computed to summarize the central tendencies and variability of all key variables. Pearson correlation coefficients were calculated to examine the bivariate relationships among general environmental beliefs, responsibility, values, and marine pro-environmental behavior. Independent-sample t-tests and one-way ANOVAs were also conducted to examine group differences where relevant.

To test the hypothesized parallel mediation model, PROCESS macro (Model 4) developed by Hayes (2017) was applied to assess the independent indirect effects of marine environmental responsibility and values on the relationship between general environmental beliefs and marine pro-environmental behaviors. Bias-corrected bootstrapping with 5,000 resamples was used to generate 95% confidence intervals (CIs) for the indirect effects. Indirect effects were deemed statistically significant when the 95% confidence interval did not include zero.

### 3.4 Ethical considerations

This study adhered to ethical standards for research involving human participants. The research protocol was reviewed and approved by the Institutional Review Board of the leading research institution (Approval No.: 1041386-202407-HR-106-02). All participants voluntarily joined the study and provided written informed consent prior to participation. Data collection was conducted anonymously to protect participants’ privacy and personal information.

## 4 Results

### 4.1 Common method bias test

To examine potential common method bias, Harman’s single-factor test was conducted by including all items from the marine pro-environmental behavior, marine environmental responsibility, values, and environmental belief scales (Zhou and Long, 2004). The analysis extracted multiple factors with eigenvalues greater than 1, and the first factor accounted for less than 40% of the total variance, below the commonly accepted threshold, suggesting that common method bias was not a serious concern. These results suggest that common method bias was not a serious concern in this study, and the measurements demonstrate a solid foundation of reliability and validity.

### 4.2 Descriptive statistics

Descriptive statistics and correlation analyses were performed for the main variables, including marine pro-environmental behaviors, general environmental beliefs, marine environmental values, and marine environmental responsibility (Table 3). The mean score for marine pro-environmental behaviors was 61.15 (SD = 10.26), for general environmental beliefs was 51.42 (SD = 6.66), for marine environmental values was 42.85 (SD = 4.29), and for marine environmental responsibility was 15.75 (SD = 3.19). Correlation analyses revealed that marine pro-environmental behaviors were significantly and positively correlated with general environmental beliefs (*r* = 0.24, *p* < 0.001), marine environmental values (*r* = 0.55, *p* < 0.001), and marine environmental responsibility (*r* = 0.79, *p* < 0.001). Moreover, general environmental beliefs were positively correlated with both marine environmental values (*r* = 0.44, *p* < 0.001) and marine environmental responsibility (*r* = 0.41, *p* < 0.001). Marine environmental values were also positively correlated with marine environmental responsibility (*r* = 0.46, *p* < 0.001). These results indicate varying degrees of positive associations among the core variables, with the strongest association observed between marine pro-environmental behaviors and marine environmental responsibility.

**Table 3:** Correlation analysis among main variables (*N* = 1,206 respondents).

Variable	*M*	SD	General environmental beliefs	Marine pro-environmental behaviors	Marine environmental values	Marine environmental responsibility
General environmental beliefs	51.42	6.66	1			
Marine pro-environmental behaviors	61.15	10.26	0.24***	1		
Marine environmental values	42.85	4.29	0.44***	0.55***	1	
Marine environmental responsibility	15.75	3.19	0.41***	0.79***	0.46***	1

***p < 0.001.

### 4.3 Hierarchical regression analyses

To examine the proposed parallel mediation model, hierarchical multiple regression analyses were conducted with marine pro-environmental behaviors as the dependent variable, controlling for gender and age (Table 4). Standardized scores of environmental beliefs, ocean environmental responsibility, and ocean value orientation were entered in successive steps. General environmental beliefs significantly predicted marine pro-environmental behaviors (*β* = 0.235, SE = 0.028, *t* = 8.362, *p* < 0.001), explaining for 5.8% of the variance in behavior [*R*^2^ = 0.058, *F*(3, 1,202) = 24.71, *p* < 0.001]. This finding confirms H1, indicating that stronger beliefs are associated with higher engagement in marine pro-environmental behaviors. When ocean environmental responsibility was added into the model, it was positively associated with marine pro-environmental behaviors (*β* = 0.312, SE = 0.03, *t* = 10.4, *p* < 0.001), and the effect of general environmental beliefs on behavior was reduced but remained significant (*β* = 0.142, SE = 0.026, *t* = 5.462, *p* < 0.001). This indicates a partial mediating role of responsibility, consistent with H2. After entering responsibility and value orientation, the explained variance substantially increased to 67.8% [*R*^2^ = 0.678, ΔR^2^ = 0.620, *F*(5,1,200) = 506.22, *p* < 0.001]. Responsibility (*β* = 0.693, *p* < 0.001) and value orientation (*β* = 0.199, *p* < 0.001) emerged as strong and significant predictors, while the direct effect of general environmental beliefs remained smaller but significant (*β* = 0.08, *p* < 0.001). These results support H2 and H3.

**Table 4:** Hierarchical regression predicting marine pro-environmental behaviors.

Predictor	*β*	SE	*t*	*p*
Step 1: Controls				
Gender	0.042	0.019	2.211*	0.027
Age	0.037	0.018	2.056*	0.04
Step 2: Beliefs only				
General environmental beliefs	0.235	0.028	8.362***	<0.001
Step 3: Adding Responsibility				
Marine environmental responsibility	0.312	0.03	10.4***	<0.001
General environmental beliefs	0.142	0.026	5.462***	<0.001
Step 4: Adding Value Orientation				
Marine value orientation	0.298	0.031	9.613***	<0.001
General environmental beliefs	0.151	0.027	5.593***	<0.001

β = standardized coefficients; SE = standard error. *p < 0.05, ***p < 0.001.

A comparison of standardized indirect effects revealed no significant difference in the mediating strength of oceanic responsibility (*β* = 0.098) and value orientation (*β* = 0.093). The contrast test for indirect effects confirmed equivalence between the two mediators (Δ*β* = −0.018), supporting Hypothesis 4 by affirming parallel and independent mediation pathways. Additionally, neither gender (*β* = 0.022, *p* = 0.191) nor age (*β* = 0.014, *p* = 0.397) significantly influenced marine pro-environmental behaviors, indicating their role was limited to statistical control. Collectively, these regression findings provide robust empirical support for Hypotheses 1–4, underscoring both direct and parallel indirect pathways through which environmental beliefs drive marine pro-environmental behaviors among Chinese university students.

### 4.4 Test of mediation effects

To further validate the parallel mediation model, mediation analyses were conducted using the PROCESS macro (Version 4.1) for SPSS (Hayes, 2017), specifying Model 4, which estimates independent indirect effects operating in parallel. A bootstrap procedure with 5,000 resamples was applied to obtain bias-corrected 95% confidence intervals (BC CIs), which offer robust estimates even under non-normality. Age and gender were entered as covariates, and all continuous variables were standardized to facilitate interpretation.

**Figure 2:**
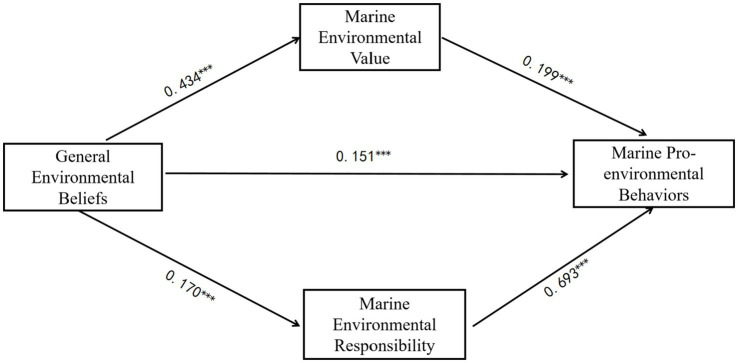
Results of a parallel mediation model. Standardized coefficients shown; ***p < 0.001. Standardized path coefficients (β) for the parallel mediation model. Unstandardized estimates (B) and bootstrap confidence intervals are detailed in Table 5.

The total effect of environmental beliefs on marine pro-environmental behaviors was significant (*B* = 0.235, SE = 0.028, *t* = 8.36, *p* < 0.001, 95% CI [0.18, 0.29]). After accounting for mediators, the direct effect remained significant but reduced (*B* = 0.080, SE = 0.018, *t* = 4.34, *p* < 0.001, 95% CI [0.044, 0.116]). The corresponding standardized coefficient was *β* = 0.151. This pattern confirms partial mediation. The indirect effect via ocean environmental responsibility was significant (*B* = 0.069, 95% BC CI [0.028, 0.110]). This path corresponds to standardized coefficients of *β* = 0.170 for Belief → Responsibility and *β* = 0.693 for Responsibility → Behavior. Similarly, the indirect effect via ocean value orientation (*B* = 0.086, 95% BC CI [0.065, 0.110]). The standardized coefficients for these paths were *β* = 0.434 for Belief → Value and *β* = 0.199 for Value → Behavior. The total indirect effect was 0.155 (95% BC CI [0.106, 0.205]). A contrast test between the two indirect effects showed no significant difference (ΔB = −0.018, 95% BC CI [−0.06, 0.027]), confirming that responsibility and value orientation operate as independent, parallel mediators (see Table 5 and Figure 2). For clarity, Figure 2 now reports the standardized coefficients (β), which correspond directly to the regression results in Table 3.

**Table 5:** Parallel mediation effects of general environmental beliefs on marine pro-environmental behaviors.

Effect type	Pathway	*B*	BootSE	95% BC CI
Total effect	Beliefs → Behaviors	0.235	0.028	[0.18, 0.29]
Direct effect	Beliefs → Behaviors	0.08	0.018	[0.044, 0.116]
Indirect effect	Via responsibility	0.069	0.021	[0.028, 0.11]
Indirect effect	Via value orientation	0.086	0.012	[0.065, 0.11]
Total indirect effect	Responsibility + Value orientation	0.155	0.025	[0.106, 0.205]
Contrast	Responsibility zishu – Value orientation	−0.018	0.022	[−0.06, 0.027]

B = unstandardized coefficients. BootSE = bootstrap SE; 95% BC CI = bias-corrected CI (5,000 resamples; Hayes, 2017). Model via SPSS PROCESS (*N* = 1,206). Effects significant if CI excludes zero. Table reports unstandardized direct and indirect effects.

Together, these findings offer strong empirical support for H2–H4, demonstrating that general environmental beliefs promote marine pro-environmental behaviors both directly and indirectly through two parallel motivational pathways—responsibility and value orientation. This model underscores the coexistence of cognitive and moral mechanisms in shaping marine conservation actions among Chinese university students.

## 5 Discussion

This study examined the mechanisms through which general environmental beliefs, responsibility, and value orientations shape marine pro-environmental behaviors among Chinese university students, and validated a parallel mediation model (PROCESS Model 4). The findings indicate that general environmental beliefs not only directly predict marine pro-environmental behaviors but also exert indirect influences through both responsibility and value orientations. The comparable mediating strength of these two pathways, stable even after controlling for demographic variables, offers novel insights for environmental psychology and marine education.

### 5.1 Interpretation of findings and psychological mechanisms

First, the results confirm the applicability of the Value–Belief–Norm (VBN) theory to marine contexts. Beliefs have long been regarded as the cognitive foundation of environmental behavior (Stern, 2000). Our findings demonstrate that the general environmental Beliefs (measured by the NEP Scale) significantly predict marine-specific attitudes (values, responsibility) and behaviors. This is a novel contribution, showing that a general environmental worldview is a strong and transferable predictor within the specialized marine context, suggesting that efforts to foster pro-environmental behavior in marine settings can benefit from focusing on general ecological principles. This study extends prior work by showing that environmental beliefs motivate behavior through two independent yet complementary mechanisms: Responsibility represents normative obligations to society and nature (the “ought to do” dimension); Value orientations capture more enduring motivational priorities (the “want to do” dimension). This dual mediation highlights that marine pro-environmental behaviors is shaped by a multidimensional motivational system, consistent with Hayes (2017) interpretation of parallel indirect pathways rather than serial mediation.

Second, the finding that responsibility and values exert nearly equivalent effects is theoretically significant. Traditionally, environmental value orientations have been viewed as more stable and dominant predictors than context-dependent responsibility (Fransson and Gärling, 1999). However, in this study, both mediators demonstrated comparable strength, suggesting a contextually specific equilibrium. One plausible explanation lies in China’s educational and cultural context, where national curricula simultaneously emphasize moral responsibility and value education under the “ecological civilization” framework (Ministry of Education of the People’s Republic of China, 2020). This institutional emphasis likely fosters a synchronized internalization of both normative obligations and biospheric–altruistic values, diminishing the expected hierarchy between them.

From a cross-cultural psychology perspective (Markus and Kitayama, 2010), the coexistence of collective responsibility and individual value endorsement reflects collectivist socialization patterns. Chinese students may integrate moral duty (toward society and nature) with personal ecological identity, unlike in European and North American contexts where value orientations often dominate behavior formation (Tam and Chan, 2017).

Finally, demographic variables such as gender and age did not significantly predict marine pro-environmental behaviors. Although previous research has identified demographic effects on environmental attitudes (Xiao and Buhrmann, 2019), the relative homogeneity of university students and their shared exposure to standardized moral and environmental education may have attenuated such differences, highlighting the stronger influence of psychological mechanisms on behavior formation.

### 5.2 Comparison with existing studies

These findings engage in meaningful dialog with prior work in environmental psychology and marine education. On one hand, they support the belief–value–responsibility framework proposed by Stern and Dietz (1994). Whereas most studies have tested this framework in the context of general terrestrial behaviors (e.g., recycling, energy saving), this study provides empirical evidence for its relevance to marine-specific behaviors in a Chinese sample. Whereas Jefferson et al. (2021) and McKinley et al. (2023) found that marine knowledge and values jointly promote conservation engagement in European and North American settings, our results indicate that in China, both responsibility and value pathways contribute equally—suggesting that collectivist cultural systems may balance normative and value-based motivations.

Furthermore, our findings challenge a common assumption in European and North American research traditions that internalized values are stronger predictors of environmental behavior than socially anchored norms (Fransson and Gärling, 1999). In contrast, Chinese participants’ strong sense of shared moral obligation—amplified through national education campaigns—appears to elevate responsibility to the same predictive level as personal values. This pattern aligns with findings from Salazar-Sepúlveda et al. (2023), who emphasized that the effectiveness of environmental interventions is conditioned by cultural context. The equivalence observed here may be a product of China’s state-guided educational ethos, in which moral cultivation and ecological awareness are jointly emphasized. Collectivist orientations, reinforced by institutional messaging, thus foster a dual motivational system—both “I should” and “I want”—that promotes pro-environmental engagement. This resonates with cross-cultural psychology perspectives (Markus and Kitayama, 2010), underscoring the need for culturally sensitive interpretations of marine pro-environmental behaviors.

Finally, the study supports psychological distance theory (Van Valkengoed et al., 2023): beliefs, when filtered through responsibility and value orientations, reduce perceived distance from marine issues and enhance behavioral relevance. This may be especially relevant for inland students, whose engagement stems not from direct marine dependence but from psychological connection shaped by moral and educational narratives.

### 5.3 Theoretical contributions and interdisciplinary significance

Theoretically, this study substantiates a parallel mediation model in which responsibility and value orientations act as independent psychological pathways linking beliefs to marine pro-environmental behavior. First, by validating a dual-pathway model of responsibility and value orientation in the marine behavioral context, this study extends the Value–Belief–Norm (VBN) theory beyond general terrestrial environmental actions to ocean-specific domains (Stern, 2000; De Groot and Steg, 2009). The finding that responsibility and values exhibit comparable mediating effects challenges the conventional assumption that value orientations are the more stable and dominant drivers of environmental behavior (Fransson and Gärling, 1999). Instead, the results indicate that normative obligations (“ought to act”) and intrinsic value-based motivations (“want to act”) may operate in parallel, together shaping pro-environmental engagement in marine settings.

Second, this research highlights the cultural embeddedness of environmental psychology. The balanced effects of responsibility and values among Chinese university students reflect the joint influence of collectivist orientations and national educational policies that promoting both moral development and ecological civilization (Markus and Kitayama, 2010; Ministry of Education of the People’s Republic of China, 2020). In contrast, studies in European and North American contexts have consistently found that self-transcendent values—such as universalism, benevolence, and personal moral norms—serve as dominant predictors of pro-environmental action (Stern and Dietz, 1994; Schultz and Zelezny, 1999; Gifford and Nilsson, 2014). These findings suggest that value-driven individual autonomy characterizes environmental engagement in European and North American societies, whereas in collectivist settings like China, pro-environmental behavior may stem from a shared moral obligation and an orientation toward social harmony (Tam and Chan, 2017; Chen and Martens, 2022). This cultural configuration explains the absence of a dominant value pathway in the present model and aligns with the Confucian ideal of the unity of knowledge and action, which views moral awareness and ecological behavior as inseparable aspects of responsible citizenship (Li and Wei, 2023).

Finally, the study holds interdisciplinary significance. In psychology, it contributes to the growing body of research on multi-pathway motivational mechanisms underlying behavior change. In education, it provides empirical grounding for the design of marine curricula that combine normative and value-oriented learning approaches, promoting both moral responsibility and emotional connection to the ocean. In marine science and policy, the findings identify university students as a key leverage group for advancing the United Nations Decade of Ocean Science for Sustainable Development (2021–2030) by fostering a generation of environmentally responsible ocean stewards (IOC Executive Council and Intergovernmental Oceanographic Commission, 2018; McKinley et al., 2023). Taken together, these contributions emphasize the importance of cross-disciplinary and cross-cultural collaboration in cultivating ocean stewardship through the integration of psychological, educational, and socio-cultural pathways.

### 5.4 Practical implications

The findings of this study carry several important practical implications for marine education, policy-making, and community engagement. First, the evidence that both responsibility and value orientation serve as independent yet equally strong mediators underscores the need to integrate normative (responsibility-based) and affective (value-based) elements into ocean literacy programs. Rather than relying solely on factual or cognitive knowledge transfer, educational interventions should cultivate students’ moral obligation and intrinsic motivation to protect the ocean (Jefferson et al., 2021; IOC Executive Council and Intergovernmental Oceanographic Commission, 2018). Experiential and participatory learning approaches—such as project-based courses, coastal clean-ups, citizen science, and virtual reality (VR) marine simulations—can help bridge the belief–action gap by strengthening both ecological responsibility and emotional connection to the ocean (Chen et al., 2025; Fauville et al., 2024). Second, policy-makers and educators should adopt strategies that connect global marine challenges (e.g., plastic pollution, climate change, biodiversity loss) to students’ local social and cultural contexts. By reducing psychological distance and increasing the perceived relevance of ocean issues to everyday life, such interventions can transform abstract environmental beliefs into actionable commitments (Van Valkengoed et al., 2023; Wang et al., 2025a,b). Moreover, policy communication should incorporate collectivist framing, emphasizing “shared ocean responsibility” and collective stewardship to align personal values with societal and national goals. Third, universities and local communities should collaborate to establish youth-led marine initiatives that translate environmental beliefs into sustained behavioral engagement. Examples include service-learning programs, marine protection clubs, and social media advocacy projects, which allow students to apply pro-environmental values through real-world participation (Ardoin et al., 2020; McKinley and Fletcher, 2010). Such programs not only internalize responsibility and values through practice, but also resonate with international goals under the UN Decade of Ocean Science for Sustainable Development (2021–2030) by cultivating the next generation of ocean stewards (IOC Executive Council and Intergovernmental Oceanographic Commission, 2018). Collectively, these findings advocate for a multi-dimensional educational and policy framework that combines cognitive awareness, moral responsibility, and emotional value orientation.

### 5.5 Limitations and future research directions

Despite its contributions, this study has several limitations. First, the cross-sectional design restricts causal inference, making it difficult to determine the temporal order of beliefs, responsibility, values, and marine pro-environmental behaviors. Future research could employ longitudinal designs or cross-cultural comparisons to test whether this parallel structure holds across cultures with different degrees of collectivism. Including emotional connectedness, perceived efficacy, and policy literacy would further refine understanding of belief–behavior dynamics. Second, the sample was drawn from universities in mainland China, which may limit the generalizability of the findings. Given cultural differences in environmental responsibility and value orientation, cross-cultural research is needed to examine the applicability of the dual-mediation model in diverse contexts. Third, the reliance on self-reported questionnaires raises the possibility of social desirability bias; incorporating behavioral observations or multi-source data would help improve measurement validity. In addition, the current model did not include other potentially influential factors such as emotional connectedness to nature, environmental efficacy, or social norms, which future studies could integrate to enrich the framework. Finally, it would be valuable to extend the research beyond university students to different age and occupational groups, and to employ educational interventions to test how responsibility and values dynamically shape marine pro-environmental behaviors. Such efforts could provide stronger theoretical and practical guidance for promoting ocean literacy and sustainable marine governance.

## 6 Conclusion

This study investigated how general environmental beliefs shape marine pro-environmental behaviors among Chinese university students, focusing on the parallel mediating roles of marine environmental responsibility and marine environmental value orientation. The findings demonstrated that beliefs not only directly predict behavior but also indirectly influence engagement through two independent and equally strong pathways—responsibility and values. Theoretically, these results extend the Value–Belief–Norm (VBN) framework to the marine environmental context, illustrating that pro-environmental behavior is driven by both normative obligations (“ought to do”) and value-based motivations (“want to do”).

Beyond theoretical advancement, the study offers practical implications for marine education and environmental policy. Integrating both responsibility-oriented and value-oriented approaches into ocean literacy programs can strengthen students’ moral commitment and intrinsic motivation toward sustainable marine behavior. Policymakers can further promote ocean stewardship by framing environmental engagement as a shared social responsibility, reinforcing alignment between individual values and collective ecological goals.

In conclusion, this research contributes to a deeper cross-cultural understanding of the cognitive–motivational mechanisms underlying marine conservation behavior. By highlighting the parallel operation of normative and affective pathways, it underscores the importance of designing educational and policy interventions that cultivate both responsibility and values. Collectively, these findings offer evidence-based directions for promoting ocean literacy, youth engagement, and the long-term governance of sustainable oceans.

## Data Availability

The original contributions presented in the study are included in the article/supplementary material, further inquiries can be directed to the corresponding author.

## Footnotes

1
https://www.wjx.cn
